# Trend and Predictors of the Utilization of Endoscopic Retrograde Cholangiopancreatography in Acute Pancreatitis Hospitalizations

**DOI:** 10.7759/cureus.11420

**Published:** 2020-11-10

**Authors:** Ashish Sharma, Jigisha H Rakholiya, Apoorva Madapu, Shivy Sharma, Anil Jha

**Affiliations:** 1 Internal Medicine, Yuma Regional Medical Center, Yuma, USA; 2 Internal Medicine, Mayo Clinic, Rochester, USA; 3 Internal Medicine, Huntsville Hospital, Huntsville, USA; 4 Internal Medicine, Marshfield Clinic Health System, Marshfield, USA; 5 Internal Medicine, Lawrence General Hospital, Lawrence, USA

**Keywords:** endoscopic retrograde cholangiopancreatography, gallstone, acute pancreatitis, nationwide inpatient sample (nis), observational study

## Abstract

Background

Acute pancreatitis is a sudden inflammation of the pancreas, and biliary pancreatitis remains the most common cause of acute pancreatitis. Endoscopic retrograde cholangiopancreatography (ERCP) is both a diagnostic and therapeutic invasive procedure to evaluate and treat pancreaticobiliary system diseases. ERCP is very commonly used in acute pancreatitis with coexisting acute cholangitis or biliary obstruction. There was a need for a nationwide study to evaluate ERCP utilization trends and health-care costs among acute pancreatitis patients.

Aim

We sought to determine the prevalence trend, hospitalization cost and stay, and predictors of utilization of ERCP amongst patients with acute pancreatitis.

Methods

We performed a population-based retrospective analysis of national data in adult acute pancreatitis hospitalizations. We evaluated the characteristics of the ERCP cohort, prevalence trend, and hospital utilization cost and stay using univariate analysis. Multivariable survey logistic regression analysis was performed to evaluate predictors of utilization for ERCP among acute pancreatitis hospitalization.

Results

Among 2,632,309 hospitalizations for acute pancreatitis, 49108 (1.87%) had ERCP. The prevalence trend of ERCP declined from 3.88% in 2003 to 0.97% in 2014.(p_Trend_<0.0001). Patients with ERCP were older (>55-years old) (53.01% vs 39.36%;p<0.0001), female (58.45% vs 48.04%; p<0.0001), Hispanic (16.30% vs 12.86%; p<0.0001), utilizing Medicare (40.29% vs 31.88%; p<0.0001), elective admission (8.15% vs 4.98%; p<0.0001), and with gallbladder etiology (65.98% vs 26.06%; p<0.0001). Acute pancreatitis hospitalization with ERCP had a higher cost of utilization (Cost_diff_:+$25077;p<0.0001) and mean stay (LOS_diff_:+3.5 days; p<0.0001). In regression analysis, old adults [Odds ratio(OR):1.087; Confidence interval (CI):1.008-1.173), Hispanic (OR:1.086; CI:1.019-1.156), asian (OR:1.146; CI:1.007-1.304), female (OR:1.074; CI:1.028-1.122), elective admission (OR:1.649; CI:1.524-1.785), gallbladder etiology (OR:4.437; CI:4.224-4.662), concurrent chronic pancreatitis (OR:1.643; CI:1.536-1.757), systemic inflammatory response syndrome (SIRS) (OR:1.264; CI:1.112-1.436), pleural effusion (OR:1.874; CI:1.231-2.854), and portal vein thrombosis (OR:1.646; CI:1.221-2.219).

Conclusion

In nationwide data, we have found a decreased utilization trend and higher hospital utilization cost and stay associated with ERCP. The predictors of utilization will be helpful to examine the cost-utility of ERCP, especially with the advent of acute pancreatitis treatment systems to mitigate the health care burden.

## Introduction

Gastrointestinal (GI) diseases account for significant health care utilization and spending [1]. In 2015, health care expenditures for GI conditions summed $135.9 billion, and among the 22 condition categories available, biliary tract disease was one of the top-five most expensive categories, costing about ($10.3 billion) [1]. Among the gastrointestinal diseases, acute pancreatitis is one of the most common causes of inpatient hospital admissions in the United States [2-3]. Acute pancreatitis was alone responsible for 279,000 hospital admissions, 0.7% in-hospital mortality, U.S. $2.64 billion in aggregate charges, and a three-day median hospital length of stay (LOS) in 2014 [1].

Acute pancreatitis treatment ranges from conservative medical management to more invasive treatment [3-4]. Endoscopic retrograde cholangiopancreatography (ERCP) is one of the commonly performed invasive procedures for the diagnosis and treatment of biliary and pancreatic diseases [2,4]. Both the therapeutic and diagnostic intervention capabilities of ERCP gives it an edge over other diagnostic utilities, however, ERCP utilization has been observed to be affected by the newly emerging diagnostic studies, such as magnetic resonance cholangiopancreatography (MRCP) and endoscopic ultrasonography (EUS), along with other patient and non-patient related factors [1-2].

The primary aim of our study is to investigate the yearly utilization trend and predictors of utilization of ERCP among patients with acute pancreatitis, and the secondary outcome is to evaluate the outcomes of the hospitalizations.

## Materials and methods

Nationwide Inpatient Sample (NIS) data between January 2003 and December 2014 were obtained from the Agency for Healthcare Research and Quality's Healthcare Cost and Utilization Project. It is the publicly available all-payer inpatient care database in the US and contains discharge-level data provided by states participating in the Healthcare Cost and Utilization Project. This administrative dataset contains data on approximately 8-million hospitalizations in 1,000 hospitals chosen to approximate a 20% stratified sample of all US community hospitals, representing more than 95% of the national population. Detailed information on NIS is available at http://www.hcup-us.ahrq.gov/db/nation/nis/nisdde.jsp.

Study population

We used the 9th revision of the International Classification of Diseases, clinical modification (ICD-9-CM) code to identify adult patients admitted to the hospital with a primary diagnosis of acute pancreatitis (ICD-9-CM code 577.0). Similarly, patients who utilized ERCP were identified using ICD-9 procedure codes 51.10. We have considered acute pancreatitis as a primary diagnosis for which hospitalization occurred and have seen ERCP amongst those patients. This population does not cover post-ERCP pancreatitis or ERCP in other urgent conditions. We used ICD-9-CM codes to identify independent predictors (covariates), including the comorbidities of hypertension, diabetes mellitus, hypercholesterolemia/dyslipidemia, smoker (current/past), drug abuse, acquired immunodeficiency syndrome, alcohol abuse/dependence and withdrawal, ischemic heart diseases, and chronic pancreatitis. Similarly, we identified complications like hypercalcemia, acute renal failure, shock, systemic inflammatory response syndrome, ascites, pleural effusion, respiratory distress/failure, and portal vein thrombosis. Table 1 mentions ICD-9-CM codes for all the concurrent conditions - adults <18 years and admissions with missing data for age, gender, and race were excluded. The sample size was based on the available data.

**Table 1:** ICD-9-CM codes used in this analysis ICD-9-CM: International Classification of Diseases, clinical modification 9

Acute pancreatitis	577.0
Gallstone	574
Hypertension	401-405
Diabetes	249-250
Obesity	278.0
Hypercholesterolemia	272.0,272.1,272.2
Drug Abuse	304.X, 305.2-305.9
Alcohol Abuse	V11.3, 303.X, 305.X
Current or Past Smoker	V15.82, 305.1
Acquired Immunodeficiency Syndrome (AIDS)	042, V08
Ischemic Heart Disease	410-414
End-Stage Renal Disease	585.6
Chronic Kidney Disease	585.1-5,585.9
Alcohol Withdrawal	291.81
Chronic Pancreatitis	577.1
Complications (%)	
Hypercalcemia	275.42
Acute Renal Failure	584.5-9
Shock	785.5
Systemic Inflammatory Response Syndrome	995.9
Ascites	789.5
Pleural Effusion	511.8
Respiratory Distress and Respiratory Failure	518.8
Portal Vein Thrombosis	452

Patient and hospital characteristics

Patient characteristics of interest were age, sex, race, insurance status, and concomitant diagnoses, as defined above. The race was defined by White (referent), African American, Hispanic, Asian or Pacific Islander, and Native American. Insurance status was defined by Medicare (referent), Medicaid, Private Insurance, and Other/Self-pay/No charge. We defined the severity of comorbid conditions using Deyo's modification of the Charlson’s comorbidity index (CCI) (Table 2). Healthcare Cost and Utilization Project NIS contains data on total charges for each hospital in the databases, representing the amount that hospitals billed for services.

**Table 2:** Deyo’s modification of Charlson’s comorbidity index (CCI)

Condition	ICD-9-CM Codes	Charlson Score
Myocardial Infarction	410 – 410.9	1
Congestive Heart Failure	428 – 428.9	1
Peripheral Vascular Disease	433.9, 441 – 441.9, 785.4, V43.4	1
Cerebrovascular Disease	430 – 438	1
Dementia	290 – 290.9	1
Chronic Pulmonary Disease	490 – 496, 500 – 505, 506.4	1
Rheumatologic Disease	710.0, 710.1, 710.4, 714.0 – 714.2, 714.81, 725	1
Peptic Ulcer Disease	531 – 534.9	1
Mild Liver Disease	571.2, 571.5, 571.6, 571.4 –571.49	1
Diabetes	250 – 250.3, 250.7	1
Diabetes With Chronic Complications	250.4 – 250.6	2
Hemiplegia or Paraplegia	344.1, 342 – 342.9	2
Chronic Kidney Disease	582 – 582.9, 583 – 583.7, 585, 586, 588 – 588.9	2
Any Malignancy Including Leukemia and Lymphoma	140-172.9, 174-195.8, 200-208.9	2
Moderate or Severe Liver Disease	572.2 – 572.8	3
Metastatic Solid Tumor	196-199.1	6
Acquired Immunodeficiency Syndrome	042 – 044.9	6

Outcomes

This study's primary aim was to evaluate the characteristics of patients with ERCP in acute pancreatitis patients, yearly utilization trends, predictors of ERCP utilization, and prevalent complications amongst acute pancreatitis underwent ERCP. The secondary aim was to evaluate the outcomes like mortality, morbidity, disability (loss of function), discharge disposition, length of stay (LOS), and cost of hospitalization associated with ERCP (years 2003-2014). The comparison of disability/loss of function was investigated by All Patient Refined Diagnosis Related Groups (APR-DRGs) severity. APR-DRGs were assigned using software developed by 3M Health Information Systems (Wallingford, Connecticut), where score 1 indicates minor loss of function, 2 - moderate, 3 - major, and 4 - extreme loss of function. Morbidity is defined as the length of stay >10 days (>90 percentile) and discharges other than home (short-term hospital, skilled nursing facility, intermediate care facility). Discharge disposition was defined by discharge to home vs. non-home [5].

Statistical analysis

All statistical analyses were performed using the weighted survey methods in SAS (version 9.4; SAS Institute, Cary, North Carolina). The p-values of <0.05 were considered significant. Univariate analysis of differences between categorical variables was tested using the chi-square test. Analysis of differences between continuous variables (age, LOS, and cost of hospitalization) was tested using the paired student's t-test. Mixed-effects survey logistic regression models with weighted analysis were used to evaluate ERCP utilization predictors amongst acute pancreatitis hospitalizations during 2003-2014. We included demographics (age, gender, race), patient-level hospitalization variables (admission day, primary payer, admission type, median household income category), hospital-level variables (hospital region, teaching versus non-teaching hospital, hospital bed size), comorbidities, concurrent conditions, complications, and CCI. The goodness of fit of the model was evaluated by the c-value.

## Results

Disease hospitalizations

**Figure 1:**
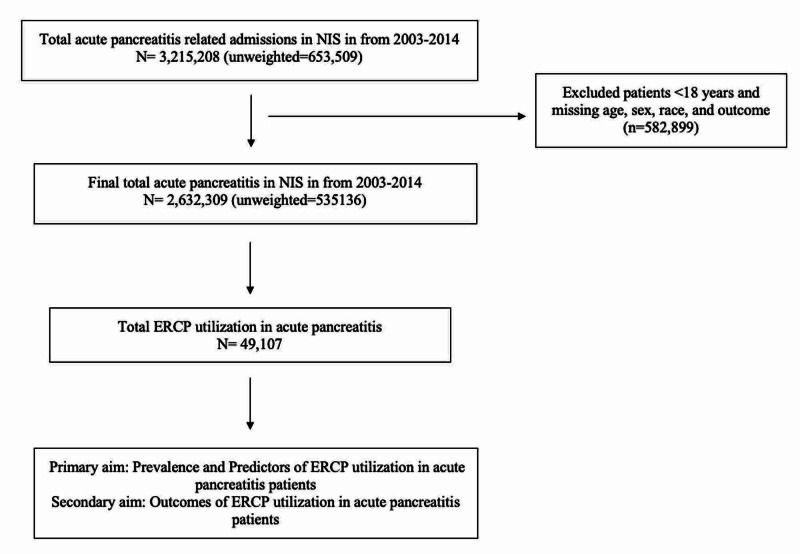
Flowchart showing details of the final study population extracted from NIS with inclusion and exclusion criteria for the study NIS: Nationwide Inpatient Sample

We found a total of 2,632,309 hospitalizations (unweighted:535,136) due to acute pancreatitis from 2003 to 2014 after excluding patients aged <18 years and admissions with missing data for age, gender, and ethnicity. Out of 2,632,309 acute pancreatitis hospitalizations, the prevalence of ERCP utilization was 49,107 (1.87%) (Figure 1).

Prevalence trend

**Figure 2:**
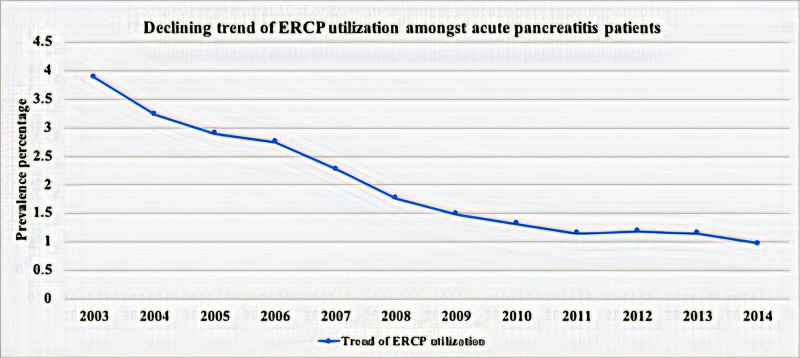
Utilization trend of ERCP amongst hospitalized patients with acute pancreatitis ERCP: endoscopic retrograde cholangiopancreatography

We analyzed the trend of utilization of ERCP in acute pancreatitis hospitalizations. As shown in Figure 2, the trend of utilization of ERCP is decreasing from 2003 to 2014 (utilization of ERCP, 3.88% in 2003 to 0.97% in 2014; p-trend<0.0001).

Demographics, patient and hospital characteristics, and comorbidities

The utilization of ERCP was more common in older age groups (mean age 57 vs 52; p<0.0001). ERCP utilization was more in patients in the age group >55 years than the age group 18-55 years (2.50% vs 1.45%; p<0.0001). Amongst the patients with acute pancreatitis who underwent ERCP, females were more likely than males (2.26% vs 1.50%; p<0.0001) and Asian and Pacific Islanders as compared to white, African American, Hispanic and native American (2.60% vs 1.88% vs 1.31% vs 2.33% vs 1.29%; p<0.0001), had Medicare (2.35%; p<0.0001), had elective admissions as compared to emergency admissions (3.01% vs 1.80%; p<0.0001), admissions on weekdays as compared to weekends (1.90% vs 1.78%; p<0.0001), and in urban-teaching hospitals as compared to rural and urban non-teaching (2.20% vs 0.74% vs 1.94%; <0.0001). The prevalence of ERCP utilization is higher than those with non-ERCP utilization in females (58.45% vs 48.04%; p<0.0001), Hispanic (16.30% vs 12.86%; p<0.0001), Medicare (40.29% vs 31.88%; p<0.0001), elective admissions (8.15% vs 4.98%; p<0.0001), weekdays (75.17% vs 24.83%; p<0.0001), and urban-teaching hospital location (47.21% vs 39.94%; p<0.0001).

Comorbidities like gallstone and gallbladder diseases (65.98% vs 26.06%; p<0.0001) and ischemic heart disease (14.41% vs 12.10%; p<0.0001) were having a higher utilization of ERCP than those with non-ERCP utilization. Comorbidities like hypertension (49.25% vs 50.45%; p<0.0001), diabetes (22.36% vs 25.93%; p<0.0001), hypercholesterolemia (7.52% vs 10.22%; p<0.0001), drug abuse (2.47% vs 6.06%; p<0.0001), alcohol abuse (9.72% vs 28.26%; p<0.0001), current or past smoker (19.25% vs 31.02%; p<0.0001), acquired immunodeficiency syndrome (0.46% vs 0.67%; p<0.0001), end stage renal disease (1.23% vs 1.65%; p<0.0001), chronic kidney disease (3.46% vs 3.95%; p<0.0001), alcohol withdrawal (0.49% vs 3.72%; p<0.0001), and chronic pancreatitis (11.77% vs 13.43%; p<0.0001) were having a lower utilization of ERCP than those with non-ERCP utilization. Complications like shock (1.03% vs 0.71%; p<0.0001), systemic inflammatory response syndrome (3.60% vs 2.51%; p<0.0001), ascites (3.07% vs 2.57%; p<0.0001), pleural effusion (0.29% vs 0.11; p<0.0001), respiratory distress and failure (3.33% vs 2.94%; p<0.0001), and portal vein thrombosis (0.50% vs 0.30%; p<0.0001) were higher among ERCP utilization than those with non-ERCP utilization (Table 3).

**Table 3:** Characteristics of patients with endoscopic retrograde cholangiopancreatography (ERCP) in acute pancreatitis hospitalizations * Bed size of the hospital indicates the number of hospital beds, which varies depending on hospital location (Rural/Urban), teaching status (Teaching/Non-Teaching), and Region (Northeast/Midwest/Southern/Western) The percentage in bracket is column % indicates the direct comparison between ERCP vs. Non-ERCP amongst acute pancreatitis patients.

	ERCP	Non-ERCP	Total	p-value	
Acute Pancreatitis Weighted (%)	49107 (1.87)	2583202 (98.13)	2632309 (100)	<0.0001	
Demographics of Patients					
Age Mean (SD) (Years)	57+/-18	52+/-17		<0.0001	
Age Group (Years)				<0.0001	
18-55 years	23076 (46.99)	1566559 (60.64)	1589635 (60.39)		
>55 Years	26032 (53.01)	1016642 (39.36)	1042674 (39.61)		
Gender (%)				<0.0001	
Male	20403 (41.55)	1342264 (51.96)	1362667 (51.77)		
Female	28704 (58.45)	1240808 (48.04)	1269512 (48.23)		
Race (%)				<0.0001	
White	32294 (68.19)	1681834 (66.99)	1714128 (67.01)		
African American	5792 (12.23)	436980 (17.41)	442772 (17.31)		
Hispanic	7718 (16.30)	322971 (12.86)	330689 (12.93)		
Asian or Pacific Islander	1286 (2.72)	48193 (1.92)	49479 (1.93)		
Native American	269 (0.57)	20548 (0.82)	20817 (0.81)		
Characteristics of Patients					
Median Household Income Category for Patient's Zip Code (%)				<0.0001	
0-25th percentile	13871 (28.88)	801293 (31.83)	815164 (31.77)		
26-50th percentile	12130 (25.25)	659970 (26.21)	672100 (26.20)		
51-75th percentile	11826 (24.62)	578597 (22.98)	590423 (23.01)		
76-100th percentile	10205 (21.25)	477773 (18.98)	487978 (19.02)		
Primary Payer (%)				<0.0001	
Medicare	19750 (40.29)	821389 (31.88)	841139 (32.04)		
Medicaid	6632 (13.53)	420525 (16.32)	427157 (16.27)		
Private Insurance	17265 (35.22)	859345 (33.35)	876610 (33.39)		
Other/Self-pay/No Charge	5378 (10.97)	475215 (18.44)	480593 (18.30)		
Admission type (%)				<0.0001	
Non-Elective	44991 (91.85)	2449856 (95.02)	2494847 (94.96)		
Elective	3990 (8.15)	128454 (4.98)	132444 (5.04)		
Admission Day (%)				<0.0001	
Weekday	36914 (75.17)	1910348 (73.95)	1947262 (73.98)		
Weekend	12193 (24.83)	672854 (26.05)	685047 (26.02)		
Characteristics of Hospitals					
Bed Size of the Hospital (%) *				<0.0001	
Small	4445 (9.07)	401046 (15.59)	405491(15.47 )		
Medium	13369 (27.27)	715471 (27.82)	728840 (27.81)		
Large	31209 (63.66)	1455584 (56.59)	1486793 (56.72)		
Hospital Location & Teaching Status (%)				<0.0001	
Rural	2844 (5.80)	379024 (14.74)	381868 (14.57)		
Urban Non-Teaching	23035 (46.99)	1165889 (45.33)	1188924 (45.36)		
Urban Teaching	23144 (47.21)	1027187 (39.94)	1050331 (40.07)		
Hospital Region (%)				<0.0001	
Northeast	11498 (23.41)	513005 (19.86)	524503 (19.93)		
Midwest	7164 (14.59)	443519 (17.17)	450683 (17.12)		
South	20093 (40.92)	1090298 (42.21)	1110391 (42.18)		
West	10353 (21.08)	536379 (20.76)	546732 (20.77)		
Comorbidities of Patients (%)					
Hypertension	24184 (49.25)	1303135 (50.45)	1327319 (50.42)	<0.0001	
Diabetes	10982 (22.36)	669914 (25.93)	680896 (25.87)	<0.0001	
Obesity	5234 (10.66)	279294 (10.81)	284528 (10.81)	0.2778	
Hypercholesterolemia	3691 (7.52)	264066 (10.22)	267757 (10.17)	<0.0001	
Drug Abuse	1215 (2.47)	156616 (6.06)	157831 (6.00)	<0.0001	
Alcohol Abuse	4775 (9.72)	730103 (28.26)	734878 (27.92)	<0.0001	
Current or Past Smoker	9455 (19.25)	801391 (31.02)	810846 (30.80)	<0.0001	
Gallstone	32400 (65.98)	673263 (26.06)	705663 (26.81)	<0.0001	
Acquired Immunodeficiency Syndrome	223 (0.46)	17389 (0.67)	17612 (0.67)	<0.0001	
Ischemic Heart Disease	7075 (14.41)	312548 (12.10)	319623 (12.14)	<0.0001	
End-Stage Renal Disease	604 (1.23)	42515 (1.65)	43119 (1.64)	<0.0001	
Chronic Kidney Disease	1698 (3.46)	102163 (3.95)	103861 (3.95)	<0.0001	
Alcohol Withdrawal	241 (0.49)	96142 (3.72)	96383 (3.66)	<0.0001	
Chronic Pancreatitis	5780 (11.77)	346851 (13.43)	352631 (13.40)	<0.0001	
Complications (%)					
Hypercalcemia	176 (0.36)	15825 (0.61)	16001 (0.61)	<0.0001	
Acute Renal Disease	3407 (6.94)	183301 (7.10)	186708 (7.09)	0.1763	
Shock	504 (1.03)	18211 (0.71)	18715 (0.71)	<0.0001	
Systemic Inflammatory Response Syndrome	1769 (3.60)	64887 (2.51)	66656 (2.53)	<0.0001	
Ascites	1506 (3.07)	66273 (2.57)	67779 (2.57)	<0.0001	
Pleural Effusion	143 (0.29)	2868 (0.11)	3011 (0.11)	<0.0001	
Respiratory Distress/Failure	1637 (3.33)	76012 (2.94)	77649 (2.95)	<0.0001	
Portal Vein Thrombosis	245 (0.50)	7791 (0.30)	8036 (0.31)	<0.0001	
Deyo's Charlson Comorbidity Index (CCI)				<0.0001	
0	27293 (55.58)	1373428 (53.17)	1400721 (53.21)		
1	12326 (25.10)	703107 (27.22)	715433 (27.18)		
2	4755 (9.68)	255546 (9.89)	260301 (9.89)		
3	2168 (4.41)	112035 (4.34)	114203 (4.34)		
4	1282 (2.61)	62336 (2.41)	63618 (2.42)		
5	1284 (2.61)	76749 (2.97)	78033 (2.96)		

Regression model derivation for predictors of ERCP utilization

In multivariate regression analysis, old adults (aOR:1.09; 95%CI:1.01-1.17; p=0.0306), male (aOR:1.07; 95%CI:1.03-1.12; p=0.0015), Hispanic (aOR:1.09; 95%CI:1.02-1.16; p=0.0104), Asian or Pacific Islander (aOR:1.15; 95%CI:1.01-1.30; p=0.0391), medium bed size of hospital (aOR:1.61; 95%CI:1.49-1.74; p<0.0001), large bed size of hospital (aOR:1.86; 95%CI:1.73-2.00; p<0.0001), urban non-teaching hospital (aOR:2.67; 95%CI:2.43-2.94; p<0.0001), urban teaching hospital (aOR:2.98; 95%CI:2.71-3.28; p<0.0001), gallstone and gallbladder diseases (aOR:4.44; 95%CI:4.22-4.66; p<0.0001), and chronic pancreatitis (aOR:1.64; 95%CI:1.54-1.76; p<0.0001) have higher odds of utilization of ERCP.

We have noticed a lower chance of ERCP utilization amongst African Americans (aOR:0.87; 95%CI:0.81-0.93; p<0.0001), other/self-pay/no charge (aOR:0.76; 95%CI:0.69-0.82; p<0.0001), weekend (aOR: 0.94; 95%CI:0.90-0.99; p=0.0134), hypertension (aOR:0.93; 95%CI:0.89-0.98; p=0.0042), diabetes (aOR:0.88; 95%CI:0.83-0.93; p<0.0001), obesity (aOR:0.81; 95%CI:0.76-0.87; p<0.0001), hypercholesterolemia (aOR:0.80; 95%CI:0.74-0.87; p<0.0001), drug abuse (aOR:0.81; 95%CI:0.70-0.92; p=0.0016), alcohol abuse (aOR:0.54; 95%CI:0.50-0.58; p<0.0001), current or past smoker (aOR:0.80; 95%CI:0.76-0.84; p<0.0001), end stage renal disease (aOR:0.70; 95%CI:0.58-0.85; p=0.0004), chronic kidney disease (aOR:0.73; 95%CI:0.65-0.83; p<0.0001), and alcohol withdrawal (aOR:0.38; 95%CI:0.28-0.52; p<0.0001) than non-ERCP utilization.

ERCP utilization was linked with having higher odds of complications like SIRS (aOR:1.26; 95%CI:1.11-1.44; p=0.0003), pleural effusion (aOR:1.87; 95%CI:1.23-2.85; p=0.0034) and portal vein thrombosis (aOR:1.65; 95%CI:1.22-2.22; p=0.0011). The c-statistic, which is used to validate the accuracy of the regressions, was 0.77 (>0.5), which indicates a good model (Table 4).

**Table 4:** Predictors of endoscopic retrograde cholangiopancreatography (ERCP) utilization in acute pancreatitis hospitalizations UL: upper limit; LL: lower limit

	Odds Ratio	Confidence Interval 95%		p-value
		LL	UL	
Age (Years)	1.00	1.00	1.01	0.0017
18-55 years	Reference			
>55 years	1.09	1.01	1.17	0.0306
Gender				
Female	Reference			
Male	1.07	1.03	1.12	0.0015
Race				
White	Reference			
African American	0.87	0.81	0.93	<0.0001
Hispanic	1.09	1.02	1.16	0.0104
Asian or Pacific Islander	1.15	1.01	1.30	0.0391
Native American	1.01	0.76	1.34	0.9394
Median Household Income Category for Patient's Zipcode				
0-25th percentile	Reference			
26-50th percentile	0.98	0.93	1.04	0.5778
51-75th percentile	0.97	0.91	1.03	0.3402
76-100th percentile	0.88	0.83	0.94	0.0002
Primary Payer				
Medicare	Reference			
Medicaid	0.97	0.89	1.05	0.4185
Private Insurance	1.03	0.97	1.10	0.2828
Other/Self-pay/No charge	0.76	0.69	0.82	<0.0001
Admission type				
Non-elective	Reference			
Elective	1.65	1.52	1.79	<0.0001
Admission day				
Weekday	Reference			
Weekend	0.94	0.90	0.99	0.0134
Bed size of the hospital				
Small	Reference			
Medium	1.61	1.49	1.74	<0.0001
Large	1.86	1.73	2.00	<0.0001
Hospital Location & Teaching Status				
Rural	Reference			
Urban Non-teaching	2.67	2.43	2.94	<0.0001
Urban Teaching	2.98	2.71	3.28	<0.0001
Hospital Region				
Northeast	Reference			
Midwest	0.75	0.70	0.81	<0.0001
South	0.87	0.82	0.92	<0.0001
West	0.79	0.74	0.85	<0.0001
Comorbidities of Patients				
Hypertension	0.93	0.89	0.98	0.0042
Diabetes	0.88	0.83	0.93	<0.0001
Obesity	0.81	0.76	0.87	<0.0001
Hypercholesterolemia	0.80	0.74	0.87	<0.0001
Drug Abuse	0.81	0.70	0.92	0.0016
Alcohol Abuse	0.54	0.50	0.58	<0.0001
Current or Past Smoker	0.80	0.76	0.84	<0.0001
Gallstone	4.44	4.22	4.66	<0.0001
Acquired ImmunoDeficiency Syndrome (AIDS)	0.85	0.60	1.20	0.3567
End-Stage Renal Disease	0.70	0.58	0.85	0.0004
Chronic Kidney Disease	0.73	0.65	0.83	<0.0001
Ischemic Heart Disease	1.02	0.96	1.09	0.5800
Chronic Pancreatitis	1.64	1.54	1.76	<0.0001
Alcohol Withdrawal	0.38	0.28	0.52	<0.0001
Complications				
Hypercalcemia	0.72	0.51	1.02	0.0630
Acute Renal Failure	0.92	0.84	1.01	0.0660
Shock	1.02	0.80	1.30	0.8618
Systemic Inflammatory Response Syndrome	1.26	1.11	1.44	0.0003
Ascites	1.13	1.00	1.28	0.0507
Pleural Effusion	1.87	1.23	2.85	0.0034
Respiratory Distress/Failure	0.93	0.82	1.06	0.2899
Portal Vein Thrombosis	1.65	1.22	2.22	0.0011
Deyo's Charlson Comorbidity Index (CCI)	1.01	0.99	1.03	0.5537
Area Under the ROC Curve/C-Index	0.77			

Other outcomes

Table 5 has mentioned the outcomes of ERCP utilization amongst acute pancreatitis patients. The patients with ERCP utilization in acute pancreatitis had a high prevalence of morbidity (8.81% vs 3.34%; p<0.0001), mortality (1.10% vs 0.97%; p=0.0037), major/extreme disability (44.69% vs 29.43%; p<0.0001), discharge other than home (17.90% vs 12.82%; p<0.0001), higher mean length of stay (8 days vs 5 days; LOSDiff=+3 days; p<0.0001), and cost of hospitalization ($56,337 vs $31,260; CostDiff=+$25,077; p<0.0001) than non-ERCP utilization.

**Table 5:** Outcomes of endoscopic retrograde cholangiopancreatography (ERCP) utilization in acute pancreatitis hospitalizations * Discharge other than home: discharge to short term hospital, skilled nursing facility, or intermediate care facility, # Morbidity defined as the length of stay >10days (>90 percentile) and discharge other than home. The percentage in bracket is column % indicates a direct comparison between ERCP vs. Non-ERCP amongst acute pancreatitis patients.

	ERCP	Non-ERCP	Total	p-value
Acute Pancreatitis weighted (%)	49107 (1.87)	2583202(98.13)	2632309 (100)	<0.0001
Morbidity#	4325 (8.81)	86328 (3.34)	90653 (3.44)	<0.0001
Mortality	541 (1.10)	25107 (0.97)	25648 (0.97)	0.0037
Disability				<0.0001
Minor/Moderate disability	26692 (55.31)	1796651 (70.57)	1823343 (70.29)	
Major/Extreme disability	21568 (44.69)	749259 (29.43)	770827 (29.71)	
Discharge Disposition				<0.0001
Discharge to home	39491 (82.10)	2154529 (87.18)	2194020 (87.09)	
Discharge other than home*	8608 (17.90)	316695 (12.82)	325303 (12.91)	
Length of Stay ± SE (Days)	8 ± 0.10	5 ± 0.01		<0.0001
Cost of Hospitalization ± SE ($)	56337 ± 866	31260 ± 76		<0.0001

## Discussion

Our study analyzed the national inpatient trend, demographics, and outcomes of acute pancreatitis patients who utilized ERCP. Besides mortality and morbidity, we have evaluated the predictors of utilization and cost of hospitalization. To the best of our knowledge, this is the first large‐scale study evaluating the trends and predictors of utilization of ERCP in this patient population. In our analysis, we observed a decreasing trend of ERCP utilization overall in patients with acute pancreatitis from 2003 to 2014. Mehta et al. have previously done a similar study, which showed a similar decreased overall trend of diagnostic utilization of ERCP, but the study specifically looked at utilization of ERCP in decompensated cirrhosis where it showed an increasing trend [6]. A recent population-based study by Somashekar et al. showed an increased prevalence of acute pancreatitis hospitalization (2002-2012). Another finding of this study was a declining frequency of acute pancreatitis with a gallstone-related disorder and increased acute pancreatitis hospitalization in association with chronic pancreatitis [7]. Our study did not look at the prevalence trend of gallstone-related acute pancreatitis; however, ERCP utilization was more associated with gallbladder etiology. The finding is interesting, as even though the prevalence of acute pancreatitis has increased, the ERCP trend has been consistently decreasing over the years. The reason for the decreasing trend of overall utilization for ERCP could be the increasing use of noninvasive diagnostic modalities of MRCP and endoscopic ultrasound [8].

Our result showed the increased utilization of ERCP in the female and old age population. Increasing age and female sex have been associated with a higher frequency of gallstone diseases, explaining the increased ERCP utilization. Increased ERCP use was also seen during acute pancreatitis hospitalization for whites, Hispanic, and Asian Pacific Islander race and is consistent with the results of the prior similar studies [9-10]. Another interesting finding of the increased ERCP utilization trend was also seen in larger bed size and urban teaching hospitals as compared to the rural and urban non-teaching hospitals. A retrospective population-based study done in 2014 to examine the disparities in colorectal cancer screening and treatment by comparing subspecialists distribution in rural and urban counties showed an increased density of gastroenterologists and other specialists in urban areas per 100,000 people as compared to rural areas [11-12]. Our study finding of comparatively less ERCP in small and rural hospitals likely reflects the resource-intense procedure nature of ERCP and the existing rural-urban disparity in the density of gastroenterologists. Similarly, inadequate subspecialist and related services coverage and availability, such as trained nursing staff and anesthesia services, could be the potential reason for the less utilization of ERCP trend on weekends compared to weekdays. The large urban and rural disparity concerns the availability of subspecialist services in rural areas, which also increases the cost burden on the health care system. [13-15] However, the results are based on limited data up till 2014. The increased number of new training programs in recent years will need further studies with recent years of data to assess real-time situations.

A higher ERCP utilization association was observed in acute pancreatitis patients with ischemic heart disease and chronic pancreatitis. However, utilization in gallstone-related disease remains at the top. Previous studies have looked at the increased rate of association in acute pancreatitis and ischemic heart disease, suggesting ischemic heart disease-related chronic inflammatory state or statin and fibrates-related adverse events being the underlying mechanism [16-17]. This particular area to establish evidence of a strong association between the aforementioned conditions probably requires further studies. However, in our opinion, the finding itself is likely related to increased age group, as increased age is a commonly known risk factor for ischemic heart disease and showed increased utilization of ERCP likely because of higher case weight of acute pancreatitis in this age group. The patients who underwent ERCP procedures were also shown to have more complication-related codes registered during the hospitalization, especially SIRS, pleural effusion, and portal vein thrombosis. Higher odds of mortality and morbidity and LOS were significantly associated with patients who underwent ERCP as compared to non-ERCP patients. As the study showed a similar association with the ERCP group's complications, the data likely reflects the possibility of having more severe pancreatitis in these patients requiring further diagnostic and therapeutic evaluation with ERCP. A previous systematic survey of the prospective study suggested a higher incidence risk for morbidity and mortality with the ERCP procedure. However, in our study, the higher odds of complications in the ERCP utilization group do not establish any causal association because of the study design and make it difficult to ascertain whether these complications resulted from pancreatitis itself post procedure complications. In general, with the advancement in overall endoscopy techniques and training, ERCP has become a much safer and effective procedure overall. Many studies have proved that ERCP is a safe procedure, and a greater number of procedures are being done as an outpatient on the elective basis [12-15].

Overall, hospital utilization cost association, length of stay, and discharges other than home were higher in the ERCP utilization population than the non ERCP utilization population. Our study is one of the largest population-based cross-sectional studies to report the predictors of ERCP utilization and outcomes and complications profile of acute pancreatitis patients who underwent ERCP. Though NIS data is the largest national inpatient database with good statistical power, this study has limitations. This administrative database is obtained retrospectively by chart abstractions based on the discharge diagnosis codes, billing codes, etc. and hence is susceptible to coding errors. The diagnosis of acute pancreatitis and other complications in the NIS database are physicians documented with clinical evidence. Complications and outcomes depend on the severity of the disease, and lab values are crucial to deciding severity and are missing. We have not followed up on the patient's post-hospitalization to evaluate the disability. Due to the study's nature, we cannot establish the temporal relationship between procedure and complications, so we can only comment on complications amongst acute pancreatitis patients who utilized ERCP.

## Conclusions

This study showed a decreasing overall trend of ERCP but a higher length of stay, cost of utilization in hospital admission, and discrepancy between urban and rural utilization. One way to decrease utilization cost along with the length of stay is to avoid unnecessary procedures in cases where suspicion for biliary etiology is low and using less invasive and cheaper alternatives compared to ERCP. More studies related to decision analysis are required to evaluate the importance of specific use of ERCP and other hepatobiliary procedures in order to mitigate the healthcare cost burden.
